# JBRA Assisted Reproduction: three decades of science, innovation, and leadership in Latin America

**DOI:** 10.5935/1518-0557.20260013

**Published:** 2026

**Authors:** Paulo Franco Taitson, Maria do Carmo Borges de Souza, Roberto de Azevedo Antunes, João Batista Alcantara Oliveira

**Affiliations:** 1 Assistant Editor - JBRA Assisted Reproduction; 2 Department of Human Reproduction, Pontifical Catholic University of Minas Gerais (MG), Brazil; 3 Editorial Consultant - JBRA Assisted Reproduction; 4 Scientific Director, Fertipraxis - Rio de Janeiro (RJ), Brazil; 5 President - Brazilian Society of Assisted Reproduction; 6 Medical Director, Fertipraxis - Rio de Janeiro (RJ), Brazil; 7 Editor-in-Chief - JBRA Assisted Reproduction; 8 Clinical Researcher, Center for Human Reproduction Prof. Franco Junior - Ribeirão Preto (SP), Brazil

The *JBRA Assisted Reproduction* (JBRA) proudly celebrates three decades of uninterrupted publication, reaffirming its role in advancing, disseminating, and internationalizing scientific knowledge in reproductive medicine. Alongside the maturation of assisted reproduction in Brazil and Latin America, the journal has served as a trusted platform for original research, clinical updates, and scholarly debate, while maintaining a longstanding commitment to editorial quality and scientific rigor (Souza et al., 2019).

Throughout its trajectory, JBRA has followed—and actively helped shape—the discussion of major scientific and clinical developments in assisted reproduction. These include refinements in IVF/ICSI practice, advances in cryopreservation strategies, preimplantation genetic testing, fertility preservation, and the growing integration of multidisciplinary care in complex reproductive scenarios. Importantly, progress in reproductive medicine depends not only on technological innovation but also on continuous critical appraisal of evidence and outcomes, as well as sustained attention to the ethical and regulatory dimensions that accompany scientific advancement.

A major milestone in JBRA’s consolidation was its indexing in international bibliographic databases, which enhanced discoverability and strengthened the global circulation of scientific output from Brazil and the broader Latin American region (Souza et al., 2019). In particular, indexing in PubMed represented a pivotal step in expanding the journal’s international visibility, ensuring that contributions from Latin America became readily searchable and accessible within the most widely used biomedical database (Souza *et al*., 2019). In a publishing ecosystem where indexing is closely linked to visibility, citation pathways, and scientific exchange, this achievement contributed to amplifying regional research in a field historically dominated by major traditional publishing centers (Souza *et al*., 2019).

JBRA’s sustained growth relies on robust editorial processes, especially peer review that is thorough, constructive, and transparent. Evidence suggests that the rigor of peer review is closely associated with the reliability and scientific contribution of published literature (Severin *et al*., 2023). High-quality peer review is therefore not merely a procedural requirement, but a core mission: to support clinically meaningful, methodologically sound, and ethically responsible research (Severin *et al*., 2023).

Although bibliometric indicators are frequently used to assess influence and reach, they must be interpreted with caution, particularly when employed as proxies for article-level quality or clinical relevance (Kondziolka, 2024). Even so, the progressive strengthening of JBRA’s international standing—reflected in citation-based metrics and ranking systems—illustrates the cumulative impact of consistent editorial work and sustained engagement from the scientific community (Souza *et al*., 2019; Kondziolka, 2024). More recently, JBRA achieved an impact factor of 1.9, signaling its expanding international reach and the increasing uptake of its published research (Kondziolka, 2024; Souza *et al*., 2019).

JBRA also plays a central role in valuing regional scientific production and fostering collaborative initiatives that characterize assisted reproduction in Latin America. Regional registry efforts, for example, generate essential large-scale data on practice patterns and outcomes, enabling benchmarking and supporting context-specific interpretation of results (Zegers-Hochschild *et al*., 2025). Such initiatives strengthen evidence generation in the region and reinforce the importance of inclusive and representative scientific visibility (Zegers-Hochschild *et al*., 2025).

Celebrating JBRA’s 30th anniversary therefore represents both recognition and responsibility: recognition of the collective efforts of editors, reviewers, authors, and readers who have consolidated a credible and respected scientific platform; and responsibility to continue advancing quality, ethical leadership, and international engagement— while remaining attentive to Latin America’s clinical realities, research priorities, and opportunities for innovation. As editorial leadership evolves, JBRA remains firmly committed to rigorous methodology, transparent editorial practices, and the publication of research relevant to patient care and health policy, while sustaining critical reflection on how scientific quality is produced, evaluated, and communicated (Kondziolka, 2024; Severin *et al*., 2023).
